# Biobanking and Regenerative Medicine: An Overview

**DOI:** 10.3390/jcm7060131

**Published:** 2018-05-31

**Authors:** David T. Harris

**Affiliations:** Department of Immunobiology, Arizona Health Sciences Centre, University of Arizona, Room 6122, P.O. Box 245221, Tucson, AZ 85721, USA; davidh@email.arizona.edu; Tel.: +1-520-626-5127

Regenerative medicine and tissue engineering play significant roles in the treatment of currently intractable conditions such as chronic heart failure, stroke, chronic osteoarthritis, and other maladies. Regenerative medicine and tissue engineering generally depend on the utilization of stem cells to treat patients but may also utilize mature cells that would not normally be considered as stem cells (e.g., skin). Stem cells (like mature cells) may be obtained from many sources in the body including bone marrow, cord blood, cord tissue, adipose tissue, etc. Although stem cells are often used in therapy immediately upon isolation, in many circumstances the stem and progenitor cells will be harvested, processed and banked frozen until a later time. Biobanking is a convenient alternative to same-day therapeutic use, in that it allows for patient recovery (e.g., from liposuction or surgery), provides time to identify the best treatment options, and may allow for multiple interventions without additional patient inconvenience or risk. 

This Special Issue will address the topic of biobanking and how it fits into regenerative medicine. Topics such as stem cell banking (e.g., cord blood, cord tissue, bone marrow, adipose tissue methodology), utilization of biobanked stem cells in pre-clinical and clinical trials, and mature cell biobanking and utilization in animal models and clinical trials (e.g., cardiomyocytes and blood vessels) will be described. Special emphasis will be put upon the role that biobanking plays in clinical therapy, precision medicine and “big data”. 

The establishment of biobanks has its origin in the laboratories that established repositories of tumors and other cell lines at the turn of the last century. These facilities were generally designed for local research use, although occasionally cell lines might be shared between laboratories on a limited basis. With the advent of stem cell transplantation for the treatment of blood borne cancers such as leukemia, it became commonplace to harvest and bank “back-up” bone marrow in case of treatment failure. These biobanks were once again local in nature, and generally not maintained for more than a couple of years at a time. As the specimens were patient-related, in general the samples were not shared between investigators. As the national research enterprise grew based on increasing federal dollars the value of well-established and well characterized cells, tissues and lines became more and more important. Upon this realization several public and private entities were created to fill this need, such as the American Type Culture Collection, the National Institutes of Health biospecimen service and the Coriell Institute. Access was often and still is limited, often requiring some form of financial remuneration/reimbursement. The discovery of stem cells in leftover umbilical cord and placental blood in the 1980s, and that it could be used in place of bone marrow for transplantation, led to the establishment and rapid expansion of stem cell banks worldwide. Over the course of the past 20 years more than 4 million cord blood samples alone have been biobanked in the US, and more than 40,000 samples have been thawed and used for transplant and regenerative medicine applications. Finally, the combined interest in precision medicine and big data, along with necessary clinical annotation of biospecimens, has led to an even greater demand for high quality, clinical grade biospecimens both for research and for clinical use in regenerative medicine and tissue engineering.

Although bone marrow banking is not routinely performed, cord blood banking for use in transplant and regenerative medicine has also led to the beginnings of cord tissue banking for future use in regenerative medicine and tissue engineering. Banking is done in the frozen state where samples can be stored indefinitely, as opposed to cold storage banks as done for red cells. For adults without access to their own cord blood collected at birth, adipose tissue banking, which is a rich source of mesenchymal stem cells (MSCs), has recently begun with reasonable success. In fact, frozen adipose tissue has been thawed after as long as 3 years in storage and used to successfully treat more than 200 patients.

Biobanking could be applied to almost any cell or tissue if proper methodology is employed. That is, it is technically feasible to freeze sheets of cardiomyocytes for cardiovascular applications, corneal limbal cells for ophthalmic applications, and endothelial cells for construction of vascular grafts. Biobanking can be advantageous in both the autologous and allogeneic settings, to reduce costs, to personalize therapies if needed, and to reduce patient inconvenience. In the autologous setting the collection and banking of biospecimens can inconvenience the patient only once, with multiple aliquots being set aside for future use. The biospecimen can be collected when the patient is at their youngest and healthiest, so that the cells are most optimal for use in therapy at any time in the future. In addition, it reduces the concerns about disease transmission and immune rejection. In the allogeneic setting it can permit selection of the most ideal biospecimen donor when personalized therapies are not needed. Young and healthy donors free of disease or other medical issues can be utilized, biospecimens expanded into hundreds if not thousands of therapeutic aliquots, and then placed at various banking sites around the country (or world) where they can be immediately available when needed. Creation of large autologous biospecimen banks (e.g., cord blood banks) can also permit clinical trial tailoring to specific patients with certain diseases or indications that shortens time to treatment, rapidly fills patient recruitment quotas and increases the probability of positive treatment outcomes. 

There is another consideration to remember concerning biobanking and regenerative medicine; precision medicine. Large scale biospecimen banking in conjunction with highly annotated clinical data for each biospecimen is crucial to identifying optimal patient demographics, therapeutic approaches for specific patient subgroups, and laying the foundation for novel discoveries based on interrogation of the big data derived from this approach. However, to protect patient identity and confidentiality it is necessary to de-identify the biospecimens. This task can be accomplished via a clinical data warehouse (CDW) using bar codes linked to patient medical record numbers (MRNs), and MRNs linked to patient electronic medical records (EMR). The biobank itself remains blind to patient identity but is able to access patient medical records and demographics. Biospecimens if collected and stored properly may be used for both therapy and research. That is, large samples such as cord blood or adipose tissue may be later removed and used for patient treatment. However, if multiple small aliquots of the specimen are also stored those “bullets” can be used for research and interrogative purposes to determine patient qualifications for trials and improved outcomes from such trials, along with providing specimens for research interrogation that produces big data that can be the source of novel discoveries and additional therapies.

In conclusion, establishment of a biobanking enterprise can be a valuable asset for regenerative medicine. The biobank can be a source of materials for therapy and for research and development. In addition, annotation of each biospecimen (in part or in whole) with relevant patient demographics and medical data can be the source of big data that leads to better patient outcomes and discovery of new therapeutic approaches. Biobanking should not be limited solely to stem and progenitor cells, as mature and differentiated cell populations can also be medically beneficial (e.g., cardiomyocytes). The biobanking approach be constrained by whether a target population is a single cell solution, as new approaches to the cryopreservation and thawing of tissues and seeded biomaterials have proven successful and increase the utility of the biobanking facility. Investment in the biobanking endeavor can have large cost recoveries as the foundation for a successful regenerative medicine and tissue engineering program.

